# Epidemiologic Pattern of Cancer in Kathmandu Valley, Nepal: Findings of Population-Based Cancer Registry, 2018

**DOI:** 10.1200/GO.20.00574

**Published:** 2021-03-31

**Authors:** Ranjeeta Subedi, Meghnath Dhimal, Atul Budukh, Sandhya Chapagain, Pradeep Gyawali, Bishal Gyawali, Uma Dahal, Rajesh Dikshit, Anjani Kumar Jha

**Affiliations:** ^1^ Nepal Health Research Council, Ramshahpath, Kathmandu, Nepal; ^2^ Environmental Health Sciences, Nepal Health Research Council, Ramshahpath, Kathmandu, Nepal; ^3^ Epidemiology, Homi Bhabha National Institute, Tata Memorial Centre, Mumbai, India; ^4^ Radiation Oncology, National Academy of Medical Sciences, Bir Hospital, Kathmandu, Nepal; ^5^ Clinical Pharmacology, Nepal Health Research Council, Ramshahpath, Kathmandu, Nepal; ^6^ Departments of Oncology and Public Health Sciences, Division of Cancer Care and Epidemiology, Queen's University, Kingston, Canada; ^7^ Homi Bhabha National Institute, Tata Memorial Centre, Mumbai, India; ^8^ Radiation Oncology, Nepal Health Research Council (NHRC), Ramshahpath, Kathmandu, Nepal

## Abstract

**PURPOSE:**

Although cancer is an important and growing public health issue in Nepal, the country lacked any population-based cancer registry (PBCR) until 2018. In this study, we describe the establishment of the PBCR for the first time in Nepal and use the registry data to understand incidence, mortality, and patterns of cancer in the Kathmandu Valley (consisting of Kathmandu, Lalitpur, and Bhaktapur districts), which comprises 10.5% of the estimated 29 million population of Nepal in 2018.

**MATERIALS AND METHODS:**

The PBCR collects information from facilities and communities through the active process. The facilities include cancer or general hospitals, pathology laboratories, hospice, and Ayurvedic centers. In the communities, the field enumerators or female community health volunteers collected the data from the households. In addition, the Social Security and Nursing Division under the Department of Health Services, which provides subsidy for cancer treatment of underprivileged patients, was another major source of data. The collected data were verified for residence, accuracy, and completeness and then entered and analyzed using CanReg5 software.

**RESULTS:**

In the Kathmandu Valley, the PBCR registered 2,156 new cancer cases with overall age-adjusted incidence rate for all cancers of 95.7 per 100,000 population (95.3 for males and 98.1 for females). The age-adjusted mortality rate for males was 36.3 (n = 365) and for females 27.0 (n = 305) per 100,000 population. We found that the commonest cancers in males were lung and stomach, whereas in females, they were breast and lung cancer. Gallbladder cancer was among the top five common cancers in both sex.

**CONCLUSION:**

These findings provide a milestone to understand the cancer burden in the country for the first time using the PBCR and will be helpful to develop and prioritize cancer control strategies.

## INTRODUCTION

Cancer is one of the major public health issues globally. It has been estimated that in 2025, more than 20 million new cases of cancer will occur in the world, majority of which would be in low- and middle-income countries (LMICs).^1^ Nepal is a low-income country in Southeast Asia, located between India and China, with a population of 26,494,504 according to the 2011 census, where cancer control has not been a priority public health agenda until recently.^2^ According to GLOBOCAN, there were an estimated 26,184 new cancer cases and 19,413 cancer deaths in Nepal in 2018,^3^ which shows cancer as a major public health problem necessitating collaborations among stakeholders to formulate cancer prevention and control strategies tailored to the country’s needs.

**CONTEXT**

**Key Objective**
To describe the population-based epidemiologic pattern, incidence, and mortality of cancer in the Kathmandu Valley, Nepal, using the first Population-Based Cancer Registry (PBCR) in the country.
**Knowledge Generated**
Among the three million population of the Kathmandu Valley, the age-adjusted incidence rate for cancer was 95.3 in males and 98.1 in females per 100,000 population with corresponding mortality rates of 36.3 and 27 per 100,000 population, respectively. The commonest cancers were lung, stomach, gallbladder, and breast (in females).
**Relevance**
This report from Nepal's first PBCR highlights the demographics and epidemiology of cancer in Kathmandu, Nepal, documenting cancer incidence, mortality, and commonest cancers. These data will be helpful to plan cancer prevention and control strategies for the government. These data support ongoing efforts to strengthen and expand PBCR throughout the country.

Cancer registries, primarily population-based cancer registry (PBCR), play a vital role in planning and implementing cancer prevention and control programs,^4-6^ documenting the scale and profile of cancer in a defined region and monitoring trends over time.^7^ Without a robust PBCR in place, data-driven cancer control strategies cannot be formulated and priorities may be misguided. A recent study summarizing the priorities for cancer control in Nepal for the next 5 years has also recommended establishment and operation of a robust PBCR as one of the top priorities.^8^

**FIG 1:**
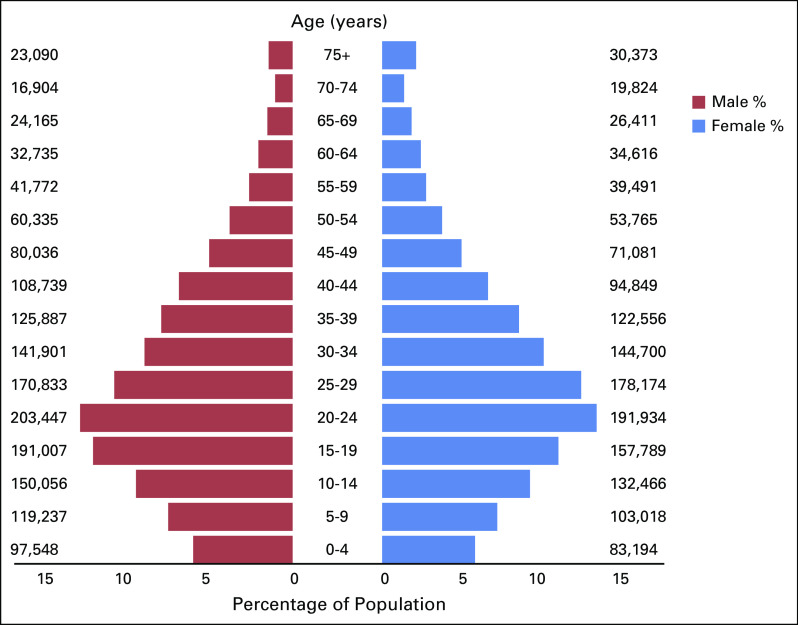
Pyramid of population distribution by age and sex in the Kathmandu Valley, 2018. The estimated population of 2018 has been calculated on the basis of 2001 and 2011 population census.

Kathmandu Valley PBCR has been initiated by Nepal Health Research Council (NHRC) since January 2018 in collaboration with the Ministry of Health and Population (MoHP), WHO, and International Agency for Research on Cancer (IARC). The registry represents the country's densely populated urban area with an estimated population of 3,071,932 (1,587,691 male and 1,484,241 female) in 2018 (Fig 1), that is, 10.5% of the national population. It comprises Kathmandu, Lalitpur, and Bhaktapur districts, with availability of diagnostic and advanced cancer treatment facilities. Using data from Kathmandu Valley PBCR, to our knowledge, this study summarizes the incidence, mortality, and patterns of cancer in the Valley for the first time in Nepal.

## MATERIALS AND METHODS

The PBCR collects data from multiple sources (hospitals, laboratories, and civil registration) as people might visit several locations for diagnosis and treatment because of lack of holistic treatment facilities, quality services, and affordability.^4^ Considering the criteria, guidelines, and protocol from the IARC and published articles, Kathmandu Valley PBCR collected primary data from all these sources and communities.

A steering committee with representatives from the MoHP and WHO and directors from major cancer hospitals was formed, which provides guidance and support to the registry, and a technical working group was formed under the steering committee. The PBCR staffs have been recruited and trained at IARC Regional Hub, India.

The Kathmandu Valley PBCR covers an area of 902 km^2^ (0.6% of the total territory, ie, 147,516 km^2^ of Nepal) with 21 urban and rural municipalities and 248 wards of Bagmati Province.^9,10^ People residing for a minimum of 6 months in these areas prior to the diagnosis of disease were considered as residents for the registry. The PBCR collects information primarily through the active process from cancer or general hospital facilities, pathology laboratories, hospice, and Ayurvedic centers that provide full or partial services to patients with cancer in the Valley. Currently, more than 28 (government or private) hospitals, three pathology laboratories, three palliative centers, and two Ayurvedic centers in the Kathmandu Valley are providing data. The two major cancer hospitals, Nepal Cancer Hospital and Research Centre (25.2%) and Bhaktapur Cancer Hospital (24.3%) share around 50% of the registry data. Because of advanced facilities, some patients with cancer (0.7%) visit B.P. Koirala Memorial Cancer Hospital, Bharatpur, the largest cancer hospital in Nepal about 250 km away from the Valley, and some visit cancer hospitals in India (0.2%). Thus, the registry collected data from these hospitals through the passive process where the focal person was oriented to send the data.

The Social Security and Nursing Division at the Department of Health Services, MoHP, is another source of registry as it has information of underprivileged patients with cancer who received financial support of up to Nepalese Rupees (NRS) 100,000 (equivalent to approximately US dollars [USD] 1,000) for treatment. Although the civil registration office is an important source of PBCR for obtaining information on cancer death cases, the PBCR in Nepal has not benefitted yet as they do not mention the cause of death as cancer in the death registry. Thus, the registry collected death records from health facilities and communities.

Community-based approach was applied coordinating with health posts in-charges, female community health volunteers (FCHVs) who worked at grassroots level in the health system of Nepal, and community leaders from each urban and rural municipality. A separate proforma was developed with some important variables to obtain preliminary information regarding cancer incidence and mortality from the household level through FCHVs. The FCHVs report collected information to health posts in-charges monthly, and health posts report to health coordinators at urban and rural municipalities. In specific wards with fewer cancer cases than estimated incidence rates, field enumerators were recruited, trained, and mobilized. The enumerators consulted FCHVs, health posts in-charges, and coordinators including ward chairperson and community leaders to identify cases in their locality and collected information through household visits.

All the collected cases were confirmed for residence and checked for accuracy and completeness. The primary site and morphology were coded using International Classification of Diseases for Oncology (3rd edition) and entered into CanReg5 software, which is an open-sourced tool to input, store, check, and analyze the data and was supported by the IARC.^11^ Certain variables (first name, last name, age at diagnosis, telephone number, and primary site) have been incorporated in the software on the basis of which it shows the list of potential duplicates after entering the data of any new cases.

Cancer cases diagnosed by all methods were registered. Borderline, in situ, and benign cases were excluded. For analysis purpose, 2018 population of the Valley was estimated using population of 2001 and 2011 census.^12,13^ The incidence and mortality according to site (International Classification of Diseases, tenth revision), age, and sex; crude rate, age-specific rates, and age-adjusted rates per 100,000 population; and cumulative risk were presented. Establishing the PBCR in resource-limited setting like Nepal is challenging where the active process of registration, paper-based recording and reporting system at many sources, and capturing death cases are the major challenges.

## RESULTS

### Incidence and Mortality Rates

The Kathmandu Valley PBCR registered a total of 2,156 new cancer cases in 2018 (male 999 and female 1,157) among the total population of 3,071,932. The overall age-adjusted incidence rate (AAR) for all cancers was 95.7 per 100,000 population (male 95.3 and female 98.1 per 100,000). Cancer deaths registered by the PBCR in 2018 were 670, of which 365 were male (Tables 1 and 2). The age-adjusted mortality rates for males was 36.3 and for females 27.0 per 100,000 population.

**TABLE 1:**
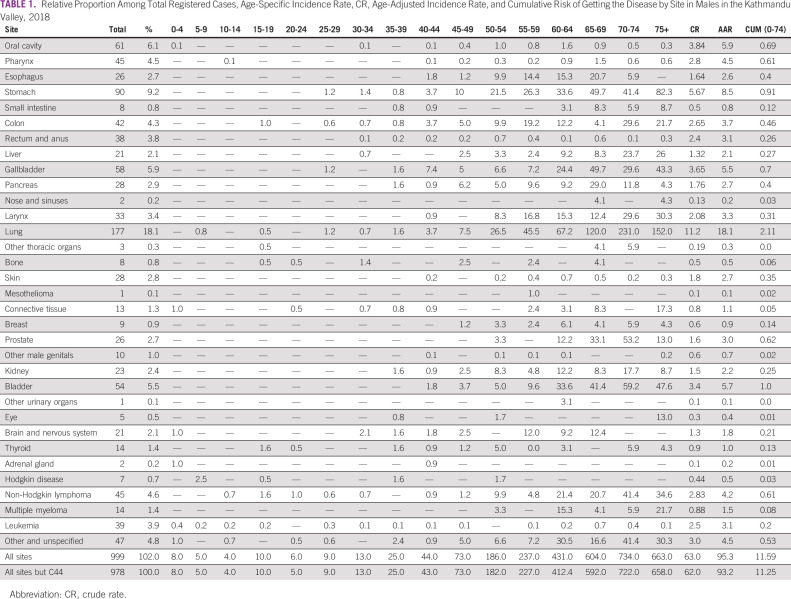
Relative Proportion Among Total Registered Cases, Age-Specific Incidence Rate, CR, Age-Adjusted Incidence Rate, and Cumulative Risk of Getting the Disease by Site in Males in the Kathmandu Valley, 2018

**TABLE 2:**
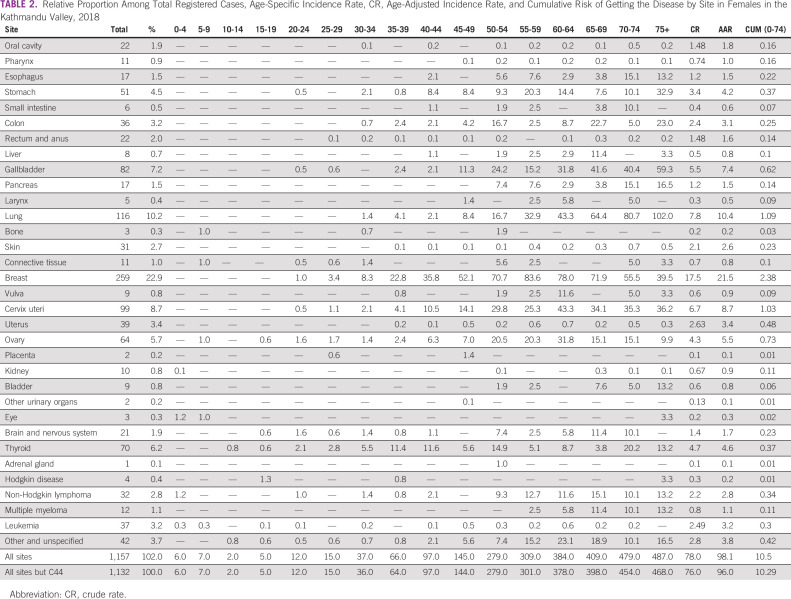
Relative Proportion Among Total Registered Cases, Age-Specific Incidence Rate, CR, Age-Adjusted Incidence Rate, and Cumulative Risk of Getting the Disease by Site in Females in the Kathmandu Valley, 2018

The incidence of cancer increased with age in both sex. In males, the peak cancer incidence was among the age group 70-74 years followed by age groups 75 years and above and 65-69 years with age-specific incidence rates (ASR) of 752.3, 584.2, and 396.4 per 100,000 population, respectively. In females, the incidence was highest among the age group 75 years and above followed by 60-64 years and 70-74 years with the ASR of 460.5, 410.2, and 396.4 per 100,000 population, respectively.

### Commonest Cancers in the Kathmandu Valley

**FIG 2:**
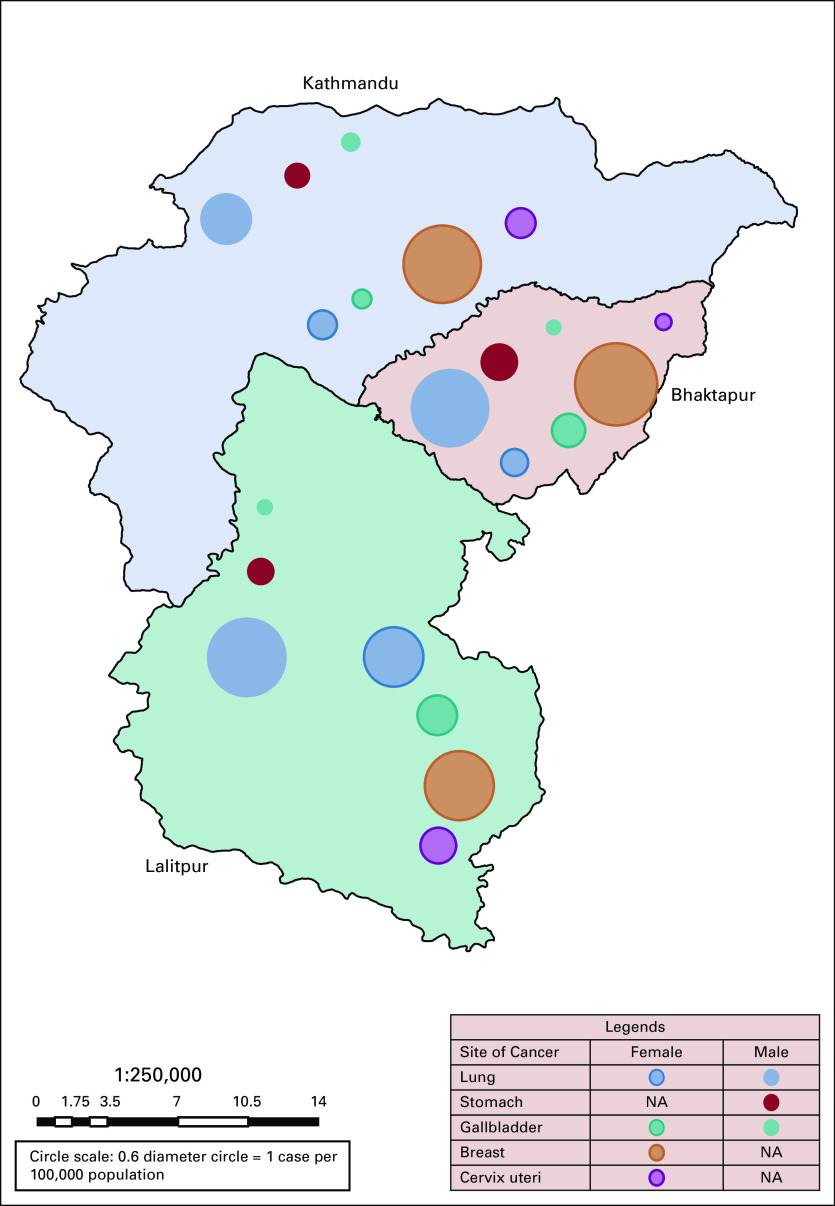
Distribution of common cancer sites on the basis of age-adjusted incidence rate within the three districts (Kathmandu, Bhaktapur, and Lalitpur) of the Kathmandu Valley, 2018. NA, not applicable.

Figure 2 shows the distribution of common cancers among males and females in three districts of the Kathmandu Valley separately. In the Valley, among males, the commonest sites of cancer were lung (18.1% of all cancers, AAR 18.1), stomach (9.2%, AAR 8.5), oral cavity (6.2%, AAR 5.8), urinary bladder (5.5%, AAR 5.7), and gallbladder (5.9%, AAR 5.5). The PBCR data of capital and major cities such as that of the Kathmandu Valley from neighboring countries China (Beijing and Shanghai) and India (Delhi, Mumbai, and Chandigarh) were compared. The AAR for cancers of the lung and oral cavity in the Kathmandu Valley were higher than those of Beijing and Shanghai in China but lower than those of Delhi, Mumbai, and Chandigarh in India. The incidence of stomach cancer in Nepal was higher compared with the cities in India but lower than the cities in China. Similarly, urinary bladder cancer incidence rates are comparable with both India and China. However, the incidence of gallbladder cancer was higher in the Kathmandu Valley than the cities of India and China (Table 3).

**TABLE 3:**
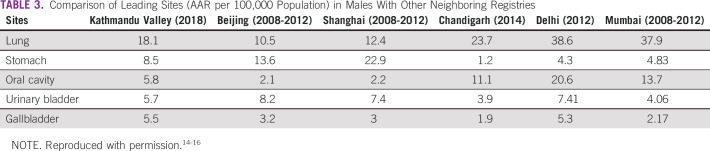
Comparison of Leading Sites (AAR per 100,000 Population) in Males With Other Neighboring Registries

The commonest sites of cancer in females were breast (22.9% of all cancers, AAR 21.5) followed by lung (10.2%, AAR 10.4), cervix uteri (8.7%, AAR 8.7), gallbladder (7.2%, AAR 7.4), and thyroid (5.7%, AAR 5.5). Among females, the incidence of breast cancer was lower in the Kathmandu Valley compared with major cities in India and China. The incidence of lung cancer in females was lower than in China but higher than in India. Similarly, the incidence of cervical cancer and gallbladder cancer was comparable with that in India; however, cervical cancer in the Kathmandu Valley was higher than that in Beijing and Shanghai in China. The incidence of ovarian cancer was found lower in the Kathmandu Valley than in urban areas of India and China (Table 4).

**TABLE 4:**
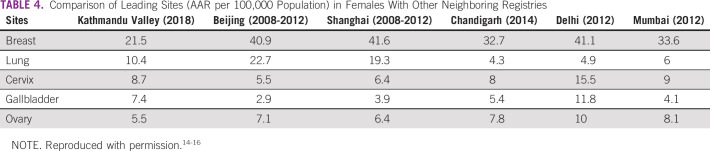
Comparison of Leading Sites (AAR per 100,000 Population) in Females With Other Neighboring Registries

Tobacco-related cancers such as cancers of the oral cavity, pharynx, larynx, lung, esophagus, pancreas, urinary bladder, and kidney accounted for a total of 32% of all cancers (653 of 2,156) in the Valley. The tobacco-related cancer cases in males was 45.8% (447) of the total male cases, whereas in females, it was 18.1% (206) of the total female cases.

Every possible effort was made to capture the 670 death cases that the PBCR registered (male 365 and female 305) in 2018. The five most fatal cancers in males were lung (24.7% of all cancer deaths, AAR 9.4), stomach (10.5%, AAR 3.7), gallbladder (6.6%, AAR 2.3), liver (5.3%, AAR 1.9), and urinary bladder (3.3%, AAR 1.7). Similarly, the most common sites of cancer mortality in females were lung (18.7% of all cancer deaths, AAR 5.4), gallbladder (13.4%, AAR 3.8), breast (11.5%, AAR 2.9), stomach (8.5%, AAR 2.2), and ovary (6.9%, AAR 1.9).

### Methods of Diagnosis

Among the 999 male cases, 901 (90%) cases were diagnosed on microscopic verification and 8% were through nonmicroscopic ways such as radiologic and clinical investigations. Among 1,157 females, 1,050 (91%) were diagnosed on microscopic verification and 7% were registered through nonmicroscopic verification. One percent of males and 2% females were registered under death certificate only cases. The death cases were registered on the basis of death certificates found in hospital files and relatives' remarks where no other information were found at hospitals during follow back.

## DISCUSSION

To our knowledge, in this study, using data from the PBCR, for the first time, we have described incidence, pattern, and mortality because of cancer in the most urban population of the country. The PBCR found that the incidence and mortality varied by age and sex with the commonest cancers being lung in males and breast in females and surprisingly high incidence of gallbladder cancers. These findings are important for planning national cancer control policy and evaluation of the ongoing intervention for cancer control. This study also reaffirms the utility of the PBCR for cancer policy planning in LMICs such as Nepal.

We found that the cancer incidence was higher but mortality lower in females versus males in the Kathmandu Valley while keeping with cancer statistics from other countries, both high- and low-income.^17^ A previous retrospective study of cancer cases collected by the Hospital-Based Cancer Registry (HBCR) from 2013 to 2020 had also showed that the cancer incidence is higher in females compared with males in all years.^18^ The PBCR from other LMICs such as Nigeria and Ghana also found higher incidence rates among females versus males.^19,20^

Lung cancer was the commonest and the most fatal cancer in both sex. Previous studies have also reported lung cancer as the major cancer among males.^18,21^ Lung cancer is associated with tobacco consumption, smoking, poor education, household air pollution, and inadequate medical health education.^22-25^ The STEPS (noncommunicable disease risk factor) survey presented that 28.9% (48.3% men and 11.6% women) were current tobacco users in Nepal,^26^ which was similar to tobacco users (28.6%) in India^27^ and tobacco smokers (27.7%) in China.^28^ The current smokeless tobacco users were 33.3% men and 4.9% women.^26^ According to the Environmental Performance Index, the air quality in Nepal is ranked 177th of 180 countries and in Asia, Kathmandu is one of the most polluted cities.^29^ Hence, smoking, tobacco use, and increasing air pollution might be considered the major risk factors of lung cancer in the Kathmandu Valley. Similarly, the burden of tobacco-related cancers was high in the Valley. Cancer of oral cavity and urinary bladder were third and fourth commonest cancer among males, respectively. The tobacco consumption was taken as one of the major risk factors of oral cancer.^30^

Although 10-year consolidated data from HBCR had previously shown cervix uteri cancer as the commonest cancer in females with breast cancer the second,^18^ the PBCR shows breast cancer was the commonest and third most fatal cancer, which is similar to that seen in the major cities of India.^31^ Furthermore, breast cancer was most common in women of 55-59 years age followed by 60-64 years and 65-69 years, in contrast to the findings of other previous studies in Nepal where 41-50 years was found to be the commonest age group for breast cancer.^32,33^ These findings will be important to plan breast cancer early-detection policies on the basis of the risk stratification.^34^

Although the etiology of breast cancer in Nepal has not been widely studied, a systematic review has found factors such as late menarche (> 14 years of age), nulliparity or late age of first birth (> 35 years), longer duration of breast feeding, smoking, excessive consumption of alcohol, and exposure to radiation as common risk factors of breast cancer in Nepal.^35^ The higher incidence of breast cancer in urban areas could have also been contributed by the adoption of westernized lifestyle and dietary pattern.^35^ The Government of Nepal has recently developed guidelines on breast cancer screening services to be provided at all levels of health facilities.^36^ Appropriate age stratification should be considered during implementation of these guidelines.

Surprisingly, cancer of gallbladder was among the top five commonest and lethal cancers in both sex, whereas it is a relatively rare cancer globally with a dismal prognosis.^37^ Some studies have shown association of history of gallstone, smoking, and early menarche with gall bladder cancer in Nepal.^37,38^ Further, locally tailored research is important to understand the reason behind the higher incidence of gallbladder cancer and identify if any preventive measures could be adopted.

Cervical cancer was the third most common cancer among females in the Kathmandu Valley with an AAR of 8.7 per 100,000 population, lower in comparison with the estimated incidence rate given by GLOBOCAN for Nepal (16.3 per 100,000 population).^3^ The lower rate in urban areas could be attributed to the improvements in lifestyle, awareness, and good hygiene practices. The incidence of cervical cancer was higher in old-age populations, being peak at age group 60-64 years, which is similar to the findings given by a hospital-based study done from 2012 to 2017.^39^ Increasing age was found as one of the risk factors of cervical cancer.^40^ Other common risk factors in developing countries include early marriage, multiple pregnancies, poor genital hygiene, multiple sexual partners, use of oral contraceptives, and lack of awareness,^40,41^ which are more common in rural areas.^42,43^

MoHP has already piloted the introduction of the human papillomavirus (HPV) vaccine among adolescents in two districts of Nepal in 2016 and has allocated budget to introduce HPV vaccination in routine immunization program.^44,45^ HPV vaccination along with screening for cervical cancer using low-cost alternatives to cervical cytology such as visual inspection with acetic acid should be the foundations of cervical cancer control in Nepal.

Because of lack of PBCR data prior to the registry, it is difficult to compare findings of the current study or assess any trends in incidence or mortality. The overall AAR (95.7 per 100,000 population) in this study was lower than the estimated rate given by GLOBOCAN for Nepal (103.7 per 100,000 population). Viewing separately, in males the AAR (87.5 per 100,000 population) is higher and in females lower (117.9 per 100,000 population) than the GLOBOCAN estimated rate.^3^ The lower rates as compared with the GLOBOCAN rate might have been caused by under-registration of the cases. However, underreporting could not be established as this is the first-year report and there is no basis for comparison.

Although it was the first year of registry, the cases registered through microscopic verification were very high showing good data quality of the registry. However, the death cases might have been underreported. Since the notification of cancer deaths is not mandatory in Nepal, NHRC is working with the Government of Nepal for policy reform on this matter. In addition, community-based approach and alternative treatment centers as the common source of data have overcome the underreporting of death cases to some extent. Besides improving data quality and coverage of cases, the registry has already established two more registries in Rukum East and West districts and Siraha, Saptari, Dhanusha, and Mahottari districts. The government has reassured its commitment to expand National PBRC across the country with remarkable increment in the funds from NRS 2,50,000 to 100,000,000 (approximately equals to 2,500-100,000 USD) from its establishment. NHRC is also actively encouraging all hospitals in the country to incorporate a HBCR so that data could be easily fed into the PBRC. In coming years, the cancer registry will also monitor the survival of patients with cancer at population level, which will be effective in monitoring cancer care.

In conclusion, in the Kathmandu Valley, the age-adjusted incidence rate was higher in males, but the age-adjusted mortality rate was higher in females. The commonest sites of cancer in males were lung, stomach, urinary bladder, gallbladder, and non-Hodgkin lymphoma, whereas those in females were breast, lung, cervix uteri, gallbladder, and ovary. Both the cancer incidence and mortality were the highest in 70-74 years age group in males and > 75 years age group in females. These findings will help the Government of Nepal and policymakers to develop cancer prevention and control strategies aligned with the cancer burden in Nepal.
